# Shared follow-up care for early breast cancer - results from an Australian national demonstration project

**DOI:** 10.1186/1472-6963-14-S2-P44

**Published:** 2014-07-07

**Authors:** Christine Giles, Caroline Nehill, Vivienne Milch, Helen Zorbas

**Affiliations:** 1 Cancer Australia, Sydney, Australia

## Background

Follow-up care after completion of breast cancer treatment is important to monitor side-effects of treatment, detect recurrence, and provide holistic support. Evidence-based best practice guidelines exist to inform the approach to the follow-up following completion of active treatment. In Australia follow-up care is carried out mostly in tertiary settings by specialist clinicians.

There are many pressures facing Australia’s health workforce, including developing technology, growing community expectations and the ageing population, which are increasing the demand for workforce services, while Australia is experiencing current and emerging shortages in the majority of medical services. It is important to ensure patient access to best-practice care is maintained across all geographic areas. New models of care are a potential solution.

## Materials and methods

Cancer Australia funded a national demonstration project to explore the acceptability to patients and health professionals, adherence to best practice and comparative costs of a shared approach to follow-up care for women with early breast cancer, across four different health service settings. The shared care model was based on the Principles of Shared Care and developed in accordance with evidence based recommendations for follow-up of women with early breast cancer.

## Results

Shared follow-up care for women with early breast cancer was demonstrated to be an acceptable model of care with agreement to participate of nearly 80% of patients approached, a much higher participation rate than similar international studies (45-67%). Best practice care was promoted with over 90% of participants receiving an individualized schedule for follow-up in line with best practice. Critical success factors were identified including clinical leadership, appropriate infrastructure, accurate and accessible electronic medical records, women having a regular GP and care coordination. Other factors that support implementation include availability of evidence-based clinical practice guidelines for follow-up, the development of tailored information and tools, and the effective focus on communication between all three important participants in the model - i.e. patient, specialist and the GP.

## Conclusions

This study demonstrated shared care to be an acceptable model of care for the follow-up of women with early breast cancer, which can improve access to care and promote the provision of care in line with best-practice recommendations. Shared care decreases the burden on specialist outpatient’s clinics resulting in a systems level approach to improving timely access to specialists for women with breast cancer. Appreciation of the critical success factors will support applicability of the shared follow-up model across other cancer groups.

